# Acute Eosinophilic Pneumonia Triggered by Carbamazepine Therapy: A Clinical and Radiologic Case Report

**DOI:** 10.7759/cureus.108598

**Published:** 2026-05-10

**Authors:** Moath Bani Salem, Shiza Virk, Yusuf Alzoubi, Ibrahim Faruqi

**Affiliations:** 1 Internal Medicine, University of Florida College of Medicine, Gainesville, USA; 2 Pulmonary and Critical Care Medicine, University of Florida, Gainesville, USA; 3 Internal Medicine, HCA Florida Blake Hospital, Bradenton, USA

**Keywords:** acute eosinophilic pneumonia, bronchoscopy, carbamazepine, eosinophilia, multifocal pneumonia

## Abstract

Acute eosinophilic pneumonia (AEP) is a type of interstitial lung disease characterized by eosinophilic infiltration of the lung parenchyma and can present as acute hypoxemic respiratory failure. Certain medications, including carbamazepine, have been rarely implicated in eosinophilic pulmonary syndromes and delayed systemic hypersensitivity reactions with pulmonary involvement.

In this case, we report a 50-year-old man who developed acute eosinophilic pneumonia (AEP) characterized by diffuse bilateral pulmonary infiltrates with small pleural effusions on imaging following the initiation of carbamazepine therapy for epilepsy approximately four weeks prior to presentation. The patient presented with fever, dyspnea, and hypoxia. Imaging demonstrated bilateral infiltrates with mediastinal and hilar lymphadenopathy. Physical examination throughout hospitalization did not reveal any rash, edema, skin desquamation, or mucosal involvement despite concern for a drug-related hypersensitivity reaction.

His clinical status worsened, leading to respiratory failure requiring intubation. Bronchoalveolar lavage (BAL) demonstrated significant eosinophilia (>25%), which was a key diagnostic finding supporting AEP. Although the patient did not meet full diagnostic criteria for DRESS syndrome, the timing of presentation and associated systemic findings raised concern for a broader carbamazepine-induced hypersensitivity reaction with predominant pulmonary involvement.

This case highlights the importance of early recognition of carbamazepine-associated eosinophilic lung disease, particularly in patients with BAL eosinophilia and compatible radiologic findings, while also emphasizing the need to evaluate for possible multiorgan involvement in suspected drug-induced AEP.

## Introduction

Acute eosinophilic pneumonia (AEP) is a rare but potentially life-threatening cause of acute hypoxemic respiratory failure [1]. AEP is characterized by the rapid onset of symptoms, diffuse pulmonary infiltrates, and eosinophilic infiltration of the lungs. Symptoms include dyspnea, fever, and cough, but may progress to acute respiratory distress syndrome requiring mechanical ventilation [2]. Because of its nonspecific presentation and diffuse pulmonary infiltrates, AEP may initially mimic community-acquired pneumonia. Many triggers have been identified, including medications such as antibiotics, nonsteroidal anti-inflammatory drugs, and neuropsychiatric agents. Among the implicated agents, carbamazepine is an uncommon but recognized cause of pulmonary hypersensitivity reactions [3].

Carbamazepine is also a well-recognized trigger for delayed T-cell-mediated systemic hypersensitivity syndromes, including drug reaction with eosinophilia and systemic symptoms (DRESS), which may involve the lungs in addition to cutaneous, hepatic, renal, hematologic, and cardiac manifestations. Pulmonary involvement may occur either as part of systemic hypersensitivity syndromes or, more rarely, as a predominantly eosinophilic pulmonary process [3].

Diagnosis of AEP depends on the identification of the offending agent, along with bronchoalveolar lavage demonstrating ≥25% eosinophils. Imaging can show bilateral ground-glass opacities, consolidation, and small pleural effusions. Peripheral eosinophilia may initially be absent early during the acute fulminant phase due to rapid eosinophil migration and sequestration within the lung parenchyma. Prognosis is generally excellent and usually responds well to discontinuation of the trigger along with systemic corticosteroids [4].

## Case presentation

In this case report, we present a 50-year-old non-smoking man with a history of ischemic cardiomyopathy and epilepsy who initially presented to our hospital with progressive shortness of breath. He had been started on carbamazepine approximately one month prior to presentation.

**Figure 1:**
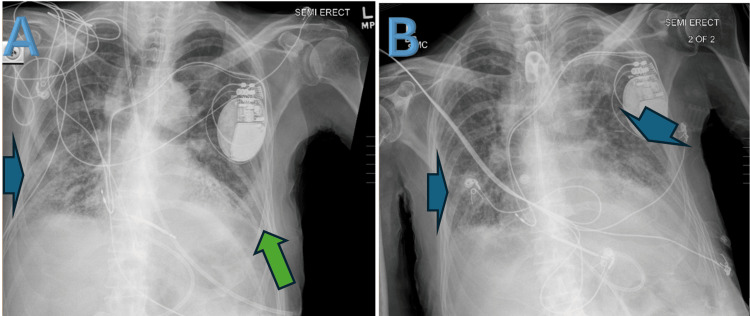
Chest X-ray Panel A showing bilateral hazy airspace opacities (blue arrow) with small pleural effusions (green arrow). Panel B showing stable mild cardiomegaly and improving pulmonary edema with increased aeration (blue arrows).

**Figure 2:**
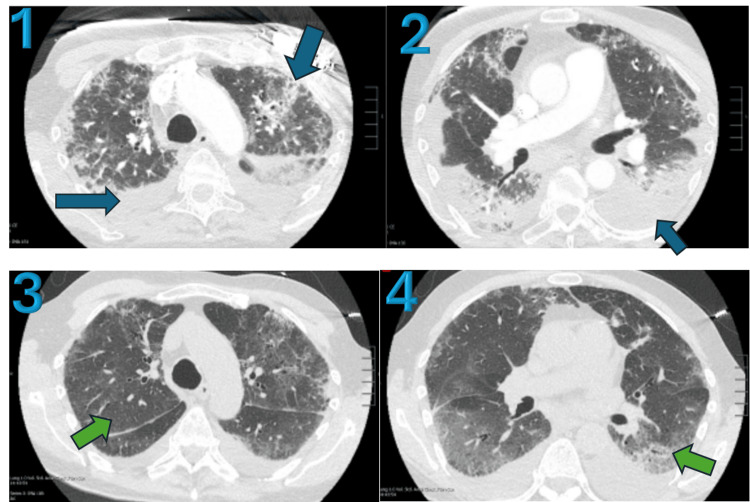
Panels 1-2: bilateral diffuse infiltrates and small bilateral pleural effusions (Blue arrows). Panels 3-4: improvement of the disease following appropriate therapy (green arrows)

On presentation, the patient had multiple febrile episodes (Tmax reaching 38.9 °C). Physical examination during hospitalization did not show any skin rash, edema, desquamation, mucosal involvement, or clinically appreciable lymphadenopathy. Laboratory and imaging studies were significant for leukocytosis of 15.4 × 10⁹/L without peripheral eosinophilia, thrombocytopenia, and acute kidney injury (Table 1). Chest X-ray showed bilateral infiltrates and a small left-sided pleural effusion (Figure 1, Panel A). Computed tomography of the chest showed bilateral diffuse infiltrates and small bilateral pleural effusions along with mediastinal and hilar lymphadenopathy (Figure 2, Panels 1-2).

**Table 1:** Comprehensive laboratory findings with reference ranges and units at presentation * Peripheral eosinophilia and absolute eosinophil count were not elevated despite significant bronchoalveolar lavage eosinophilia, supporting the diagnosis of acute eosinophilic pneumonia during the acute fulminant phase. pCO₂ = partial pressure of carbon dioxide; pO₂ = partial pressure of oxygen; HCO₃ = bicarbonate ^ Ventilator settings during arterial blood gas collection: assist-control pressure control ventilation, respiratory rate 20 breaths per minute, fraction of inspired oxygen 60%, positive end-expiratory pressure 5 cmH₂O

Category	Test	Result	Reference Range	Units
Arterial Blood Gas (ABG)^	pH	7.22	7.35–7.45	—
	pCO₂	68.8	35–45	mmHg
	pO₂	167	75–100	mmHg
	HCO₃	26.8	22–26	mmol/L
	Oxygen Saturation	99.3	95–100	%
Complete Blood Count (CBC)	White Blood Cell Count (WBC)	15.6	4.0–11.0	×10⁹/L
	Hemoglobin	10.9	13.5–17.5	g/dL
	Hematocrit	32	41–53	%
	Platelet Count	86	150–400	×10⁹/L
	Neutrophils	92.8	40–70	%
	Eosinophils	0.1	0–8.0	%
	Absolute Eosinophil Count*	0.02	0.0–0.5	×10⁹/L
Metabolic Panel	Sodium	135	136–145	mmol/L
	Potassium	4.5	3.5–5.0	mmol/L
	Glucose	188	70–99	mg/dL
	Calcium	7.7	8.5–10.5	mg/dL
	Phosphorus	5.8	2.5–4.5	mg/dL
	Albumin	3	3.5–5.0	g/dL
Renal Function	Blood Urea Nitrogen (BUN)	43	7–20	mg/dL
	Creatinine	2.15	0.6–1.3	mg/dL
	Estimated Glomerular Filtration Rate (eGFR)	31	>60	mL/min
Liver Function Tests	Aspartate Aminotransferase (AST)	24	10–40	U/L
	Alanine Aminotransferase (ALT)	70	7–56	U/L
	Alkaline Phosphatase	265	44–147	U/L
	Total Bilirubin	0.5	0.1–1.2	mg/dL
Endocrine	Hemoglobin A1c (HbA1c)	5.7	<5.7	%
	Thyroid Stimulating Hormone (TSH)	2.45	0.4–4.0	mIU/L
Microbiology	Blood Cultures	Negative	—	—
Bronchoscopy / BAL Findings	Bronchoalveolar Lavage Eosinophils	28	<25	%

The patient was initially treated for community-acquired pneumonia. However, his clinical status did not improve, prompting further evaluation with bronchoscopy and bronchoalveolar lavage (BAL), which demonstrated 28% eosinophils, with a subsequent negative infectious workup. Following bronchoscopy, his respiratory function deteriorated, leading to acute hypoxemic and hypercapnic respiratory failure requiring intubation and mechanical ventilation. His respiratory failure was felt to be multifactorial, likely related to severe AEP with a component of pulmonary edema in the setting of underlying heart failure.

Given the absence of typical environmental triggers for AEP, a drug-induced etiology was suspected. Carbamazepine was discontinued, and the patient was transitioned to levetiracetam. Systemic corticosteroids were initiated, resulting in significant clinical improvement and successful extubation. Interval imaging showed marked improvement of pulmonary infiltrates (Figure 1, Panel B, and Figure 2, Panels 3-4).

Despite initial respiratory improvement, the hospital course was complicated by ST-elevation myocardial infarction (STEMI), worsening pulmonary edema, recurrent respiratory failure requiring re-intubation, arrhythmias, worsening kidney function, and multiorgan failure. He ultimately required renal replacement therapy and tracheostomy placement. Given continued clinical decline, the patient was transitioned to comfort-focused care.

## Discussion

Eosinophilic pneumonia (EP) is a rare respiratory condition classified as a type of interstitial lung disease [1]. EP can be further divided into acute and chronic forms. Acute eosinophilic pneumonia (AEP), also referred to as idiopathic acute eosinophilic pneumonia, is a severe and rapidly progressive lung disease. It typically affects males between the ages of 20 and 40, particularly smokers without a history of allergies [2,3].

The pathophysiology of AEP is not fully understood. The current hypothesis suggests a hypersensitivity reaction to a specific exposure that is usually inhaled but may also be systemic. The body responds through eosinophil recruitment, leading to pulmonary inflammation [2]. Typical exposures include tobacco smoke, vaping, and environmental dust. Several medications have also been associated with AEP, including carbamazepine [4].

Carbamazepine is additionally well known to trigger delayed systemic hypersensitivity syndromes, particularly DRESS syndrome, which commonly presents within 2-8 weeks following drug initiation [5]. Pulmonary involvement may occur as part of these systemic reactions and can manifest as eosinophilic pneumonia, interstitial pneumonitis, or diffuse pulmonary infiltrates [6]. However, isolated eosinophilic pulmonary involvement without definite systemic hypersensitivity has also been reported [7].

In our patient, symptoms developed approximately four weeks after initiation of carbamazepine, which raised concern for a delayed hypersensitivity reaction. The patient also had recurrent fevers and lymphadenopathy on imaging. However, he did not meet diagnostic criteria for DRESS syndrome, with a RegiSCAR score of 1, and notably had no skin rash, edema, desquamation, mucosal involvement, or peripheral eosinophilia during hospitalization [5]. Although thrombocytopenia and renal dysfunction developed during the hospital course, these findings were interpreted cautiously, given the patient's prolonged ICU stay, recurrent intubations, changing hemodynamics, anticoagulation exposure, aggressive diuresis, and later need for renal replacement therapy. Therefore, the presentation was felt to be more consistent with carbamazepine-associated AEP rather than definite systemic DRESS syndrome.

The absence of peripheral eosinophilia also does not exclude AEP. In the acute phase, eosinophils may rapidly migrate and compartmentalize within the lung parenchyma, making BAL eosinophilia an important diagnostic feature [2,4]. In our patient, BAL demonstrated 28% eosinophils, supporting the diagnosis of AEP in the appropriate clinical and radiologic setting [3].

Based on the literature review, we found one case report demonstrating carbamazepine-associated AEP following dose escalation for trigeminal neuralgia [8]. Other reports have described a broader spectrum of carbamazepine-induced pulmonary toxicities ranging from eosinophilic pneumonia and interstitial pneumonitis to bronchiolitis obliterans organizing pneumonia [7,9,10]. While skin involvement remains the most common manifestation of carbamazepine hypersensitivity, severe systemic complications, including hepatitis, myocarditis, renal injury, and hematologic abnormalities, have also been reported [5,11].

An additional important aspect of this case was the later development of STEMI in a patient with pre-existing ischemic cardiomyopathy. Although hypersensitivity-associated cardiac involvement, such as Kounis syndrome or eosinophilic myocarditis, may be considered in the differential diagnosis of carbamazepine-related hypersensitivity reactions [12,13], the patient developed worsening pulmonary edema, recurrent respiratory failure, arrhythmias, and multiorgan failure in the setting of prolonged critical illness and severe underlying cardiac disease. Therefore, the cardiac complications were felt to be more likely related to critical illness and underlying ischemic cardiomyopathy rather than definite DRESS-related cardiac involvement. Corticosteroids may theoretically contribute to cardiovascular risk; however, the clinical course as well as the patient's hypoxemia were felt to be the main precipitating factors in this case.

## Conclusions

AEP is rare but can be life-threatening if diagnosis is delayed, as respiratory function may deteriorate rapidly, leading to prolonged hospitalization and increased risk of complications. Appropriate and timely management of AEP, including discontinuation of the offending agent and early initiation of corticosteroids, can dramatically reduce disease severity, shorten hospitalization, and improve overall outcomes. This case additionally highlights the importance of evaluating for systemic multiorgan hypersensitivity involvement when drug-induced AEP is suspected, particularly in patients exposed to aromatic anticonvulsants such as carbamazepine, while also recognizing that AEP may present as an isolated eosinophilic pulmonary process rather than solely as part of a broader systemic hypersensitivity reaction.
